# Facilitators and Barriers to Linkage to HIV Care among Female Sex Workers Receiving HIV Testing Services at a Community-Based Organization in Periurban Uganda: A Qualitative Study

**DOI:** 10.1155/2016/7673014

**Published:** 2016-07-14

**Authors:** Sharon Nakanwagi, Joseph K. B. Matovu, Betty N. Kintu, Frank Kaharuza, Rhoda K. Wanyenze

**Affiliations:** ^1^MakSPH-CDC Fellowship Program, Makerere University College of Health Sciences, School of Public Health, Kampala, Uganda; ^2^Reach Out Mbuya Parish HIV/AIDS Initiative, Kampala, Uganda; ^3^Department of Community Health & Behavioral Sciences, Makerere University College of Health Sciences, School of Public Health, Kampala, Uganda; ^4^Department of Disease Control and Environmental Health, Makerere University College of Health Sciences, School of Public Health, Kampala, Uganda

## Abstract

*Introduction*. While four in ten female sex workers (FSWs) in sub-Saharan Africa are infected with HIV, only a small proportion is enrolled in HIV care. We explored facilitators and barriers to linkage to HIV care among FSWs receiving HIV testing services at a community-based organization in periurban Uganda.* Methods*. The cross-sectional qualitative study was conducted among 28 HIV positive FSWs from May to July 2014. Key informant interviews were conducted with five project staff and eleven peer educators. Data were collected on facilitators for and barriers to linkage to HIV care and manually analyzed following a thematic framework approach.* Results*. Facilitators for linkage to HIV care included the perceived good quality of health services with same-day results and immediate initiation of treatment, community peer support systems, individual's need to remain healthy, and having alternative sources of income. Linkage barriers included perceived stigma, fear to be seen at outreach HIV clinics, fear and myths about antiretroviral therapy, lack of time to attend clinic, and financial constraints.* Conclusion*. Linkage to HIV care among FSWs is influenced by good quality friendly services and peer support. HIV service delivery programs for FSWs should focus on enhancing these and dealing with barriers stemming from stigma and misinformation.

## 1. Introduction

Female sex workers (FSWs) are at high risk of HIV infection and transmission. A systematic review conducted in over 25 countries with medium and high HIV prevalence indicated that 36.9% of sex workers in sub-Saharan Africa were HIV positive with some countries having HIV prevalence as high as 70.7% [1]. Despite this high burden of HIV infection, only 58% of sex workers have access to HIV prevention and care programmes [2] and less than half of HIV positive sex workers are enrolled into HIV care [3].

Antiretroviral therapy (ART) can reduce the risk of HIV transmission by 96% [4]. UNAIDS reported that, in 2013, ART averted 6.3 million AIDS related deaths worldwide as a result of the increased number of people receiving ART [5]. In 2015, WHO released revised guidelines for HIV treatment and recommended initiation of ART among all HIV infected individuals irrespective of their CD4 count, for improved treatment outcomes and prevention of HIV transmission [6]. Sex workers are among the priority populations for expansion of HIV services including ART. However, despite the existence of established targeted sex worker interventions, lots of challenges still exist that have hindered many from enrolling into care.

Several studies have highlighted barriers to linkage to care among female sex workers including discrimination by hospital staff [3, 7], delays at the health facility [8, 9], lack of money for transport and food, fear of drugs, stigma [3], use of drugs and alcohol [9], fear to be seen by clients [8], lack of knowledge about the treatment center [10, 11], and delays in accessing treatment among those who felt they were still healthy [9]. However, while these barriers have been reported, there is still limited information on linkage to HIV care among sex workers in sub-Saharan Africa, particularly on effective linkage models to inform scale up of HIV interventions among sex workers [12].

In 2012, Reach Out Mbuya HIV/AIDS Initiative (ROM), a Faith-based community non-government organization in Uganda, introduced mobile outreach services to promote HIV testing and treatment among FSWs and their clients. Although the program has over 200 FSWs enrolled in HIV care, some of the FSWs who tested HIV positive did not enrol for treatment. We conducted a study to identify the facilitators and barriers to linkage to HIV care among the FSWs who tested positive in order to design appropriate HIV interventions for this key population group.

## 2. Methods

### 2.1. Study Design

This was a cross-sectional qualitative study that involved in-depth and key informant interviews. This study was part of a larger study that explored the facilitators and barriers to linkage to HIV care among FSWs in Kampala and Wakiso districts in Central Uganda.

### 2.2. Study Site and Population

The study was conducted among FSWs accessing HIV services from ROM. ROM promotes HIV prevention through peer educators and provides HIV counselling and testing (HCT) services through established outreach clinics that provide care and treatment for HIV positive FSWs. Mobile HCT outreaches are conducted in locations surrounding Mbuya parish in Kampala and Wakiso districts in Central Uganda.

### 2.3. Selection of Interview Participants

In-depth interview (IDI) participants were selected from 301 HIV positive female sex workers who were registered in the HCT registers at the different outreach points between May 2012 and December 2013. Of the 301 registered clients, 144 were traced and interviewed as part of the larger study where 28 of these were purposively selected to participate in the in-depth interviews. These included 14 FSWs who were in HIV care and 14 who had not yet enrolled into care. The selected FSWs were contacted by counsellors by phone to explain the study and to ask them to participate in the study. FSWs with missing phone contact information or those who were unavailable after a minimum of three telephone attempts were tracked through their peer educators and friends.

Key informant interview (KII) participants were selected from peer educators who were trained by ROM and were involved in following up FSWs to encourage them to access HIV care. In addition, five ROM staffs who were involved in the implementation of the outreach program were interviewed as key informants.

### 2.4. The ROM HIV Testing and Linkage Program

ROM provides static and bi-weekly outreach clinics for FSWs using staff specifically trained on how to deal with key populations including FSWs. At the time of the study, HIV testing and linkage to HIV care services were offered using bi-weekly mobile outreach clinics, brothel based testing, and nocturnal mobile vans at places where the FSWs reside or conduct business. HIV testing was conducted following the national rapid testing algorithm [13] and posttest counselling was provided to HIV infected women who were advised on the available health facilities for follow-up care. HIV infected women were advised to begin care immediately either at the static or mobile clinics operated by ROM or other partners and were followed up with phone calls or by peer educators a week after testing and once every quarter thereafter, to ascertain linkage to care. Those who were enrolled at the static clinic were subjected to a home assessment by the Community ART and TB treatment supporters (CATTS) to enable adequate follow-up. The information collected using the home assessment tool included the names and number of household members, water and sanitation, economic status, property owned, distance to the facility, and acceptable mode for communication.

### 2.5. Data Collection and Management

**Figure 1:**
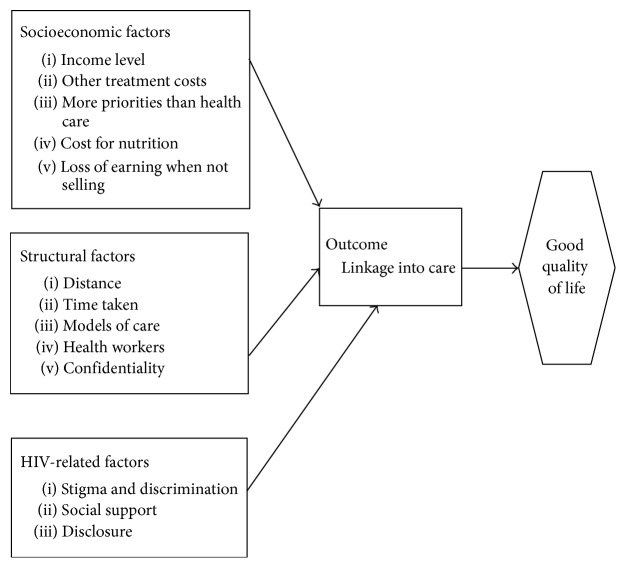
Conceptual framework for the factors influencing FSWs linkage to care.

Data collection was conducted between May and July 2014. Sex workers, who agreed to participate in the study, provided written informed consent and were interviewed in the language of their choice using unstructured, pretested questionnaires. The pretesting was conducted in the nearby project area two weeks before the commencement of the study. To ensure anonymity, participant names were replaced with initials. Data were collected on the facilitators and barriers to linkage to HIV care by trained research assistants with experience in handling FSWs. Data were collected on age, education, and linkage status to aid proper categorization of the study participants (linked or not linked to care). We defined linkage to care as having registered within an HIV clinic. The interview themes (i.e., sociodemographic, socioeconomic, structural, and HIV-related factors) were informed by theoretical constructs from the Health Belief Model [14] and the Socio-Ecological Model [15] and further informed by literature on barriers and facilitators to HIV service access among FSWs.  Figure 1 presents a summary of the key variables and their interrelationships in informing linkage to HIV care among FSWs.

Interviews were conducted at selected brothels, FSWs homes, and static and outreach clinics. The interviews were tape-recorded, translated, and transcribed verbatim.

Key informant interviews were conducted at the workplace or residence of the respondents, in English and Luganda, using unstructured pretested questionnaires. The themes for the key informant interviews included HIV treatment seeking behaviour of the FSWs, the challenges of enrolling into care, and the interventions to enhance linkage to HIV care in this population. Additional data, including age, education, job category, residence, and the time allocated to working with FSWs, was collected. The interviews were tape-recorded and later transcribed verbatim. Those that were conducted in Luganda were concurrently translated and transcribed.

### 2.6. Data Analysis

The transcripts were printed and reviewed by SN and JKBM. The review of transcripts was guided by a priori themes pertaining to linkage to care, loss to follow-up, stopping treatment, and facilitators and barriers to linkage. Issues that cut across the different themes which were captured by both authors were coded and categorized while those that were captured by one but not the other were subjected to further analysis until they were resolved and used to support the overriding themes or dropped. Data were presented using themes and backed up with quotations.

### 2.7. Ethical Considerations

The study was approved by Makerere University School of Public Health Higher Degrees Research and Ethics Committee (IRB#: IRB00011353) and the Uganda National Council of Science and Technology. Participants provided written informed consent after explanation of the study rationale and procedures. Interviews were conducted in a private setting identified in consultation with the participants.

## 3. Results

### 3.1. Participant Characteristics

Of the 28 FSWs interviewed, 14 (50%) were enrolled in HIV care (Nine at ROM), 8 had not yet enrolled, and 6 had previously enrolled but were either lost to follow-up or had stopped treatment. Of the 28 women interviewed, 24 (85%) were aged 20–30 years while the rest where >30 years. Half (14) had at least secondary or higher education and the other half (14) had at least primary education. The FSWs who were lost to follow-up or had stopped treatment were aged between 15 and 36 years with their education level ranging from none to secondary. The respondents included FSWs who operate in bars (6), on the streets (8), at home (3), and in brothels (11) (Table 1).

### 3.2. Facilitators of and Barriers to Linkage into HIV Care

The findings have been grouped into two themes: (a) facilitators of linkage to HIV care and (b) barriers to linkage to HIV care among FSWs, as presented below.

### 3.3. Facilitators of Linkage into HIV Care

The facilitators have been further grouped into two main themes: health system and social network factors (Table 2).

### 3.4. Health Systems Factors

#### 3.4.1. Good Quality Services Offered by Reach Out Mbuya

Majority of the participants associated their linkage to care with the good quality of the services including good attitude and friendliness exhibited by the counsellors towards them. They noted that the counsellors talked to them with ease and were not judgmental:

*The counsellors are good, they talk to us very well and are almost like our parents. When I was told my results I got so scared as though I would die there and then. I failed to breath for 30 minutes; the counsellor got me some cold water to drink and the care she showed me encouraged me to enrol for treatment.  (FSW, 29  years, enrolled  in  care)*

Participants reported that the counsellors provided detailed information and encouragement to help them start treatment. The counsellors told them that testing HIV positive was not the end of life and that with medication they would be helped to stay healthy and live longer. In addition, participants appreciated the good follow-up support for those who tested positive. Some said the counsellors called them and followed them up to their homes, which made them feel cared for:

*I started treatment due to counselor X's advice, she was so caring and always followed me up, she could visit me; otherwise on my own I had even disappeared but she was on my case. (FSW, 24  years, enrolled  in  care)*

Some participants acknowledged that it was easy for them to enrol in care because of the same-day results and immediate initiation of treatment. The peer educators and fellow sex workers enabled them to start treatment by encouraging them and helping to allay their fears regarding HIV treatment and occasionally escorting them to the clinics to start medication. Some respondents said they would forget the clinic days because the clinic did not run every day but the peer educators reminded them. During the key informant interviews the peer educators mentioned how tirelessly they worked to follow up the HIV positive FSWs; they were sometimes abused by the sex workers but they never gave up on them:

*I feel it is my calling to help these people and even though I am not paid I make sure I have brought them to get medicine. (peer  educator, 29  years)*

* … they come and tell me that on such and such a date I will be going to the clinic so remind me, I will note this in my diary and when the date approaches I go to them and tell them it is the day for the clinic. (peer  educator, 25  years)*

The proximity of the clinic to their residence was another enabling factor for linkage to care. Some FSWs did not want to move long distances for HIV care and others did not have transport so they thought that enrolling at ROM would help reduce such burdens.

### 3.5. Social Network Factors

Across all interviews FSWs who were enrolled in savings and other support groups mentioned advantages of joining these groups. They noted that the members of the support groups like village savings groups started by ROM for sex workers only or mixed with other community members helped them to enrol into care. The members of the savings group reportedly advised them to start on treatment and live longer. Being part of a savings group also created hope that money would be available for food and they would have capital to start their own business and live longer:

*I thought I was alone but when I realized we were many, I stopped fearing and also started medicine. My group members say that everyone is sick and so we should use our money well to care for ourselves and look nice to get customers and save much more. (FSW, 25  years, enrolled  in  care)*

Across all the interviews the participants cited the need to remain healthy as one of their motivators for linkage to care. In order to continue doing their work, they wanted to start medication to remain healthy and not show any signs of opportunistic infections, which would scare away their customers. Others wanted to remain healthy so as to live longer and take care of their children:

*It is because of my children; I have to live for them because I am a widow, if I don't take care of myself and die who will take care of them. (FSW  with  3  children, 24  years, in  care)*

Several participants reported that seeing their colleagues improve after starting treatment motivated them to start on treatment while others did so because many of their friends were dying, which compelled them to start treatment so as to survive:

*I had a friend who stopped treatment and died, when we took her to the hospital the nurses said if only she continued with treatment she would be alive. This challenged me and the next morning I had to look for a clinic. When they (providers) came looking for sex workers to be enrolled into care I was the first on the list to go for treatment. You need to take the initiative yourself since it is your life. (FSW  20  years, enrolled  in  care)*

### 3.6. Barriers to Linkage into Care

Barriers were similarly categorized into two themes: health systems and social network factors (Table 3).

### 3.7. Health Systems Factors

#### 3.7.1. Negative Attitude of the Health Workers

The FSWs enrolled at the government facilities mentioned discrimination from the health workers who openly exhibited a negative attitude towards sex workers. They said the health workers reacted differently after realizing that they were dealing with a sex worker and, upon telling their friends, those who had not yet registered for care feared to enrol:

*The counselor asked me what I do and I said sex work. She called four other counselors to ask me why I do that kind of work; I wanted to ask them, ‘what do you expect me to do?' … but the counselor stood up and asked me rudely; ‘is that also work to mention among people?' That counselor annoyed me so much I walked off and never wanted to see her again. (peer  educator  who  is  a  FSW, 29  years)*

#### 3.7.2. Rigid Treatment Policies

The four FSWs who were enrolled at government facilities noted that they were discouraged by the requirement to present a treatment supporter coupled with the long procedures for enrolment that involved several visits to the facility:

*It was not easy to start ARVs because I was told that unless I went with someone I would not be started on ARVs and this person was always moving and too hard to get. This was a challenge to me in starting treatment. I felt this was not right because I must go for treatment by myself … may be I do not want someone else to know that I am positive. (FSW, 29  years, enrolled  in  care)*

### 3.8. Social Network Factors

#### 3.8.1. Stigma

Almost all the participants cited stigma as a major barrier to linkage to care. The majority feared to be seen by their clients at the clinic. Although the close proximity of the clinic was mentioned as a motivator, for some FSWs it was a hindrance. Participants were afraid of being seen at the HIV clinic by customers, their friends, and other people in the village and were particularly concerned about rumours:

*I cannot go to that clinic because everyone here goes there and they will see me which will spoil my business. Right now the competition is much and the other girls will make me a topic because they want to take away my customers yet they are also sick. (FSW  22  years, not  enrolled)*

#### 3.8.2. Myths and Fears about ARVs

Due to lack of information, many participants had many myths about ARVs (e.g., that they kill fast and need a lot of care). They avoided the ARVs by not going to the clinic while some opted to wait until they got other jobs because of the perception that ARVs could weaken them and disrupt their work. Some respondents feared drugs and could not imagine swallowing them for life so they opted out. On the other hand, some participants feared death after testing and lost hope for living; they did not see the need for starting on ART:

*Madam isn't there any danger with the ARVs because friends tell me that when I start taking ARVs they will burn my kidney and lungs (FSW, 20  years, not  in  care  and  never  went  to  school)*

#### 3.8.3. Inadequate Information about HIV and Denial of HIV Test Results

Some FSWs did not know what HIV is while others encountered difficulties in identifying an HIV clinic because they lacked proper information about the location of the clinics. This was mainly a challenge to the young girls (aged between 20 and 25 years) who lived in the brothels as indicated by the project staff:

*Some were testing for the first time and others had ever tested but they were not even shocked when told that they were positive because they were just starting to understand what HIV is. (key  informant)*

Some FSWs mentioned that they could not enrol in an HIV clinic since they did not believe their HIV results. Some thought it was just a prick performed hurriedly in the night and wondered how it translated into an HIV positive result so they waited and retested many times:

*When I reached home, I started doubting the results and decided to go for another test in a different place. This was now the third time I was testing positive. I continued living in denial but after some time I had to admit that I was HIV positive and started taking septrin. (FSW  29 yrs, not  enrolled  in  care)*

A third of the FSWs mentioned that they were using herbs as a substitute for ARVs and therefore saw no need of being registered in an HIV clinic. This was done following negative advice from fellow sex workers. Besides many of the FSWs said they used local herbs from traditional healers to attract customers and did not have to line up for herbs like they did in the HIV clinic:

*No. I have not received treatment for HIV except local herbs which I prefer to take and I do not want to be forced to take medication [meaning ARVs]. Even with the herbs now am on medication; I do take the local herbs and am fine with the treatment, I don't want to be forced to take ARVs. (FSW, 24 yrs, not  enrolled)*

Some participants bought painkillers from general clinics instead of enrolling in an HIV clinic (self-medication). Even when the nurses in these clinics suspected that they were HIV infected, they did not tell them because they wanted business through treating their on and off sickness.

#### 3.8.4. Lack of Time due to Busy Work Schedules

Because they are always travelling in different places looking for clients, FSWs cited lack of time as one of the barriers to linkage to care:

*I am still very busy right now looking for rent and food. I cannot lose any customer at this difficult moment but when I have raised some money to care for myself I will start medicine without any worries. (FSW  23  years, not  enrolled)*

#### 3.8.5. Category of Sex Worker

The KIs felt that the street based sex workers had more challenges with linkage to HIV care compared to those in brothels because they were difficult to trace given that they only relied on phone numbers which were off most of the time:

*Those on the streets do not want to access care…. (unlike) those in the brothels (who) can come for care. They [street based FSWs] test positive…. The next day when we call their phones are off. (key  informant)*

#### 3.8.6. Financial Constraints

Across all interviews, the FSWs mentioned lack of finances as a major hindrance to linkage to care. They were concerned about requirements such as good food and frequent transport to the health facility:

*It's the financial challenges and hunger because sometimes I go without food and my friends tell me that taking medication requires eating and drinking. (FSW  22  years, not  enrolled)*

## 4. Discussion

Our study of facilitators and barriers to linkage to HIV care among female sex workers in Kampala and Wakiso districts in Uganda shows that health system and social network factors are the major facilitators and barriers to linkage to HIV care. Good quality health services (especially polite and caring providers, strong follow-up structures using peer educators, and provider telephone calls) and social network factors (encouragement from peers and membership of savings group and the need to maintain good health) were the primary facilitators of linkage to HIV care among FSWs. On the other hand, perceived stigma, various forms of misinformation, and prohibitive clinic policies were major barriers to linkage to HIV care. Contrary to the experience of FSWs at ROM, those who were registered in the public health facilities encountered negative staff attitudes, a major barrier that has been documented in several studies [8, 16, 17].

These findings suggest that efforts to improve linkage and retention in HIV care among FSWs should focus on quality of services and improving social support networks. Ensuring good quality services, particularly providers who can deliver services in a respectful and no-judgmental manner [18] without undue delays and restrictions and adequate community education and mobilization as well as support structures for retention are key health system issues that can improve the quality of services. Using peers to educate communities and follow-up of those who need treatment reduce the burden on health workers and strengthens community engagement [19]. As reported elsewhere, the requirement of a treatment supporter before initiating care and treatment was a barrier to treatment that should no longer be strictly enforced [8].

The training of providers and peer educators prior to implementation of this program could have contributed to delivery of quality services at ROM and are a key intervention area [12]. Given the overriding concerns about being seen at HIV facilities, training for providers should emphasize the need for privacy and confidentiality. Further, providing an integrated package of services for sex workers could reduce stigma around accessing HIV and other services. Training should also emphasize flexibility with certain clinic policies that could hinder access to HIV services among sex workers.

Social network issues were also strong facilitators and barriers to linkage and require interventions particularly targeted at enhancing support by peers and reducing misinformation and stigma. Enacted stigma, myths, and fears about ARVs coupled with lack of correct information about HIV continue to be major barriers to linkage to HIV care [3].

Financial constraints were a prominent barrier to linkage to care. The savings groups were a good avenue for social support and financial stability and could be integrated into health programs for low-income sex workers [20, 21]. Collectively, these findings suggest that programs targeting FSWs should integrate continuous sensitization and provision of tailor made services that address the individual level, community, and health system needs of FSW.

Our study had some limitations. This study is based on interviews with 14 FSWs who were in HIV care and 14 others who were not. By all counts, the numbers are too small to represent the views of all FSWs who are enrolled or not enrolled in HIV care. However, our study findings provide good insights into the facilitators and barriers to linkage that can inform interventions to improving linkage for FSWs. Future research is needed to further understand how the FSWs can be maintained in care once they have been linked.

## 5. Conclusion

Our study shows that FSWs ability to be linked to HIV care is largely influenced by good quality friendly services and community support systems especially from their peers. HIV programs for FSW should focus on enhancing these as well as dealing with the barriers that mainly stem from stigma, ignorance, and work-related challenges. These findings call for a need to design interventions that utilize a multichannel, multipronged approach to increase access to HIV services among FSWs.

## Figures and Tables

**Table 1:** Sociodemographic characteristics of study participants.

Characteristic	Number (*N* = 28)	Percentage
*Age-group*		
20–30	24	86
30+	04	14

*Education*		
Primary	14	50
Secondary	14	50

*Area of operation*		
Bars	06	21
Street	08	29
Home	03	11
Brothel	11	39

*Enrolment status*		
In care	14	50
Not yet enrolled	08	29
Lost to follow-up/stopped treatment	06	21

**Table 2:** Facilitators for linkage into HIV care.

Theme	Subtheme	Key emerging issues
Health systems factors	Good quality service offered by Reach Out Mbuya	(i) Good attitude and friendliness exhibited by counsellors (ii) Counsellors provided detailed information and encouragement to start treatment (iii) Good follow-up support using phone calls and home visits (iv) Same-day results and immediate initiation on treatment (v) Linkage and retention support: encouragement and reminders from peers trained by ROM (vi) Short distance to the clinic

Social network factors	Enrolment in a savings group	(i) Encouragement from group members (ii) Restored hope (iii) Money for food and capital to start business
	Need to remain healthy	(i) Continue work (ii) Avoid signs of opportunistic infections (iii) To live longer and take care of their children
	Experiences of HIV infected colleagues and friends	(i) Sick friends improving (ii) Sick colleagues dying of HIV

**Table 3:** Barriers to linkage to HIV care.

Theme	Subtheme	Key emerging issues
Health systems factors	Negative attitude of health workers Rigid treatment policies	(i) Discrimination from health workers at the government health facilities (ii) Requirement of a treatment supporter (iii) Long procedures for enrolment

Social network factors	Stigma	(i) Fear to be seen at the clinic
	Myths and fears about HIV and ARVs	(i) ARVs kill fast and need a lot of care (ii) ARVs weaken and disrupt work (iii) Fear of swallowing drugs for life (iv) Loss of hope due to fear of death after testing
	Lack of information about HIV and denial of HIV results	(i) Do not know what HIV is (ii) Difficulty in identifying an HIV clinic due to lack of information (iii) Do not believe the HIV test results (iv) Use of herbs and self-medication
	Work-related factors	(i) Busy work schedule/lack of time (ii) Travelling in different places in search for clients (iii) Difficulty in tracing the street based FSWs
	Financial constraints	(i) Lack of money for food (ii) Transport costs to the health facility
